# Systematic review and meta-analysis of balance training in children with developmental disorders

**DOI:** 10.7717/peerj.21272

**Published:** 2026-06-10

**Authors:** Yahui Nie, Nuannuan Deng, Dandan Huang

**Affiliations:** 1 College of Physical Education, Southwest University, Chongqing, China; 2 Physical Education Institute, Chongqing Technology and Business University, Chongqing, China; 3 College of Physical Education, Chongqing University, Chongqing, China

**Keywords:** Balance, Children, Cerebral palsy, Down syndrome, Disorder

## Abstract

**Background:**

Children exhibiting atypical developmental trajectories often experience motor skill impairments, making them a priority for targeted interventions. While the benefits of balance training (BT) are well-established in typically developing populations, its impact on children with developmental disorders remains underexplored.

**Objective:**

This systematic review aimed to assess the effectiveness of BT in enhancing motor performance among children with developmental or health-related conditions.

**Methods:**

A comprehensive search was conducted across PubMed, Web of Science Core Collection, CINAHL, MEDLINE, SPORTDiscus, Scopus, and CNKI, covering publications up to January 2026. Eligible studies were limited to randomized controlled trials that examined BT interventions targeting at least one motor skill (*e.g.*, balance, strength) in children with developmental disorders.

**Results:**

A total of 22 trials met the inclusion criteria. The findings indicated that BT produced moderate effects on static balance (ES = 0.90), dynamic balance (ES = 0.65), functional balance (ES = 1.01), and gross motor function (ES = 0.55). Only a limited number of studies examined muscle strength and coordination, and positive effects were reported.

**Conclusions:**

The findings support the potential of BT to enhance balance and gross motor function in children with developmental disorders. Nevertheless, the current evidence base is constrained by methodological heterogeneity, and evidence-based guidance on optimal BT protocols remains lacking. Future research should prioritize high-quality, standardized trials that investigate task-specific BT interventions and systematically examine dose–response relationships to better clarify effectiveness and support translation to real-world clinical and educational settings.

## Introduction

Childhood represents a formative stage that shapes an individual’s cognitive, emotional, social, and health trajectories (Bornstein, Hahn & Haynes, 2010; Luecken, Roubinov & Tanaka, 2013). However, children with atypical developmental patterns often face both physical and cognitive difficulties. Developmental coordination disorder (DCD), affecting approximately 5–6% of school-aged children, is a neuromotor condition characterized by poor motor coordination that impairs daily activities and school performance (Zwicker et al., 2012). Children with DCD generally require more time and support to develop motor skills compared to typically developing peers (Sylvestre et al., 2013). Cerebral palsy (CP) is a neurological disorder that impairs motor control, muscle tone, and posture, and often leads to secondary complications that further restrict functional ability over time (Patel et al., 2020). Similarly, children with Down syndrome (DS) tend to have lower birth weights and slower physical growth than their peers without DS (Zemel et al., 2015). Without timely and targeted interventions, these developmental difficulties may continue into adolescence and adulthood.

BT is a rehabilitative approach designed to improve or restore the body’s ability to maintain stability (Schedler et al., 2020). It supports postural maintenance, smooth transitions between positions, and effective responses to external disturbances (McKeon & Hertel, 2008). These exercises are frequently used to enhance physical performance and reduce injury risk. Gerards et al. (2023) investigated their impact on stability and fear of falling in older populations. Zech et al. (2010) also confirmed the benefits of such training for static and dynamic balance in both athletic and non-athletic groups. Among adolescents, Granacher, Gollhofer & Kriemler (2010) observed gains in lower-extremity muscle power, postural control, jump ability and following balance-focused interventions. Research by Al-Attar et al. (2022) indicated that balance exercises, whether performed in isolation or as part of injury prevention strategies, effectively reduce ankle injury risk. In pediatric populations, Muehlbauer (2021) found that brief interventions of four to six weeks improved both static and dynamic balance in healthy children. Furthermore, Sushma et al. (2025) reported that incorporating BT into the routines of overweight and obese children significantly enhanced postural stability.

Research on the use of BT in pediatric populations is limited, particularly among children with developmental disorders, and its effectiveness remains unclear. To date, there appears to be no systematic review that comprehensively evaluates its impact on performance in this group. Understanding the outcomes of such interventions is vital for guiding clinical decision-making and designing effective rehabilitation strategies. This review, therefore, aimed to synthesize evidence from randomized controlled trials (RCTs) investigating BT in children with developmental disorders and to examine its influence on motor performance.

## Materials & Methods

### Protocol and Registration

This review was preregistered in the PROSPERO database (CRD420251031890), conducted in accordance with the Cochrane Handbook for Systematic Reviews of Interventions, and reported following the PRISMA guidelines (Page et al., 2021).

### Literature search

The authors conducted a systematic computerized search of PubMed, Web of Science Core Collection, CINAHL, MEDLINE, SPORTDiscus, and Scopus, up to January 11, 2026. In addition, CNKI is the most comprehensive digital database for accessing scholarly literature published by Chinese researchers (Dai et al., 2024). Thus, CNKI was selected as the primary database for identifying studies published in Chinese. The search used the Boolean syntax: (“balance training” OR “balance exercise*” OR “instability training” OR “perturbation training” OR “proprioceptive training” OR “sensorimotor training” OR “Wii Fit” OR “balance board”) AND (disabilit* OR disorder* OR deficit OR “cerebral palsy” OR “Down Syndrome” OR “autism spectrum”) AND (children OR puberty OR kids OR teen* OR boy* OR girl*). To ensure comprehensive coverage, additional manual searches were carried out *via* Google Scholar and by screening the reference lists of all included studies. Detailed search strategies for seven databases are provided in Supplementary Material S1.

### Selection criteria

This review included peer-reviewed literature published in English or Chinese, as these are the authors’ working and native languages, ensuring accurate interpretation of study details and outcomes, with no restrictions on publication year. Studies were selected based on the PICOS framework (Amir-Behghadami & Janati, 2020) and had to meet the following inclusion criteria: (1) population: children aged 4–14 years with developmental disorders (*e.g.*, CP, DS, autism spectrum disorder (ASD)); (2) intervention: BT involving static postural control tasks (*e.g.*, single-leg standing or tandem stance) and/or dynamic postural stabilization exercises (*e.g.*, weight shifting, stepping, or walking-based balance tasks), with interventions comprising either a single specific type of BT or a combined form of BT (*e.g.*, balance and resistance training). In addition, studies employing exergames (or virtual reality) as the balance exercise intervention (*e.g.*, using a balance board), with outcomes assessed using validated balance or other motor performance tests rather than game-based scores, were included; (3) comparator: control groups involved daily activities, regular physical therapy or alternative training programs (*e.g.*, core training); (4) outcome: measures of balance (static, dynamic, functional) and/or other motor performance outcomes (*e.g.*, strength and coordination); and (5) study design: RCTs.

Exclusion criteria were: (1) studies involving adults or participants outside the specified mean age range; (2) exergame-based exercises not using a balance board; (3) non-randomized controlled designs; and (4) studies that did not report relevant outcomes and/or failed to provide both baseline and post-intervention measurements.

Two reviewers (ND, YN) independently screened all records by reviewing titles, abstracts, and full–text articles to determine eligibility. Disagreements were resolved through discussion, and any unresolved cases were adjudicated by a third reviewer (DH) until consensus was reached.

### Coding of studies

Data extraction was independently conducted by two authors (YN, ND) using a structured coding sheet. Any discrepancies were reviewed and resolved by a third author (DH), ensuring accuracy of the final data. The extracted variables included: (1) author, and publication year; (2) type of disorder; (3) sample size, sex distribution, and age; (4) intervention characteristics (*i.e.,* content, duration, session frequency, and session length); and (5) primary outcomes (*e.g.*, balance, gross motor performance, and strength).

### Assessment of the methodological quality and risk of bias

The quality of the RCTs was evaluated using the Physiotherapy Evidence Database (PEDro) scale, which comprises ten items assessing the methodological rigor of RCTs, including aspects such as randomization, blinding, and outcome reporting (Maher et al., 2003). The PEDro scale is known for its reliable and valid measures and is commonly utilized in physiotherapy research. Quality was categorized according to established cut-offs used in previous BT systematic reviews (Lesinski et al., 2015; Pirayeh et al., 2022; Coelho et al., 2022), with scores of ≤3 denoting poor quality, scores of 4–5 indicating moderate quality, and scores of 6–10 representing high quality. Two reviewers (YN and ND) independently assessed the studies’ quality using the PEDro scale. In parallel, the risk of bias in randomized studies was independently evaluated by two authors (YN and ND) using version 2 of the Cochrane Risk of Bias tool (RoB 2) (Sterne et al., 2019). Traffic light and weighted summary plots were generated using the online risk of bias VISualization (robvis) tool (McGuinness & Higgins, 2020). In cases of disagreement, a consensus was reached through discussion.

### Certainty of evidence

The overall certainty of the evidence was appraised using the Grading of Recommendations Assessment, Development, and Evaluation (GRADE) framework (Guyatt et al., 2011; Zhang et al., 2019a; Zhang et al., 2019b). All outcomes were initially rated as high certainty and could be downgraded by one level based on predefined criteria, including methodological limitations (mean PEDro score < 6), substantial inconsistency (statistical heterogeneity exceeding 75%), and imprecision (fewer than 400 participants per outcome). Indirectness was not downgraded, as the included studies addressed a clearly defined population, intervention, and outcome set. Potential publication bias was assessed using Egger’s regression test. For outcomes where the number of available trials was insufficient to permit meta-analysis, the certainty of evidence was rated as very low (Ramirez-Campillo et al., 2022).

### Data synthesis and analysis

A meta-analysis was performed when a minimum of three studies reported adequate data for the same outcome; if this criterion was not met, a narrative synthesis was employed instead. Means and standard deviations (SD) for a measure of pre-post-intervention performance were used to calculate between-group effect sizes (ES; Hedges’ g). Data were standardized using post–intervention SDs. If sufficient data were not reported (*e.g.*, missing or presented only in graphical form), corresponding authors were contacted to request the required information. If no response was received or the requested data could not be obtained, the outcome was excluded from the analysis. The inverse–variance random-effects model for meta-analyses was used because it allocates a proportionate weight to trials based on the size of their individual standard errors (Deeks, Higgins & Altman, 2008) and facilitates analysis while accounting for heterogeneity across studies (Kontopantelis, Springate & Reeves, 2013). Correlation values were not available in all studies; therefore, a conservative correlation of 0.5 was assumed (Follmann et al., 1992). ES are presented alongside 95% confidence intervals (CIs), and were interpreted as follows: <0.2 (trivial), 0.2–0.6 (small), 0.6–1.2 (moderate), 1.2–2.0 (large), 2.0–4.0 (very large), and >4.0 (extremely large) (Hopkins et al., 2009). In studies with multiple intervention arms, the sample size of the shared group was split according to the Cochrane Handbook (Higgins et al., 2019) and Rücker, Cates & Schwarzer (2017) to avoid “double-counting” of participants (*i.e.,* unit-of-analysis error). The control group sample size was split equally across pairwise comparisons (*e.g.*, nc*=* nc/k*), while the control mean and SD remained unchanged. Heterogeneity was assessed using the I^2^ statistic. I^2^ values of < 25%, 25–75% and > 75% were considered to represent low, moderate and high levels of heterogeneity, respectively (Higgins & Thompson, 2002). When more than 10 study groups were included, potential publication bias was assessed using funnel plot asymmetry (Cirone et al., 2023; Deng et al., 2024). When fewer than 10 study groups were available, the risk of publication bias was examined using the extended Egger’s test, with *p* < 0.05 indicating the presence of bias (Egger et al., 1997; Afonso et al., 2024). In case of a significant Egger’s test, the trim and fill method proposed by Duval and Tweedie was applied for adjustment (Duval & Tweedie, 2000). All analyses were carried out using the Comprehensive Meta-Analysis (CMA) software (version 3; Biostat, Englewood, NJ, USA).

Subgroup analyses were performed to explore potential moderator variables. Participants were categorized using a median split (Deng et al., 2023) based on training duration (≤10 weeks *vs.* > 10 weeks). In addition, the effects of BT were compared across different delivery modalities (*i.e.,* regular BT *vs.* Exergame-based BT). Subgroup analyses were conducted only when at least three studies provided data for a given moderator. Meta–regression was not performed due to the limited number of included studies.

## Results

### Study selection

**Figure 1:**
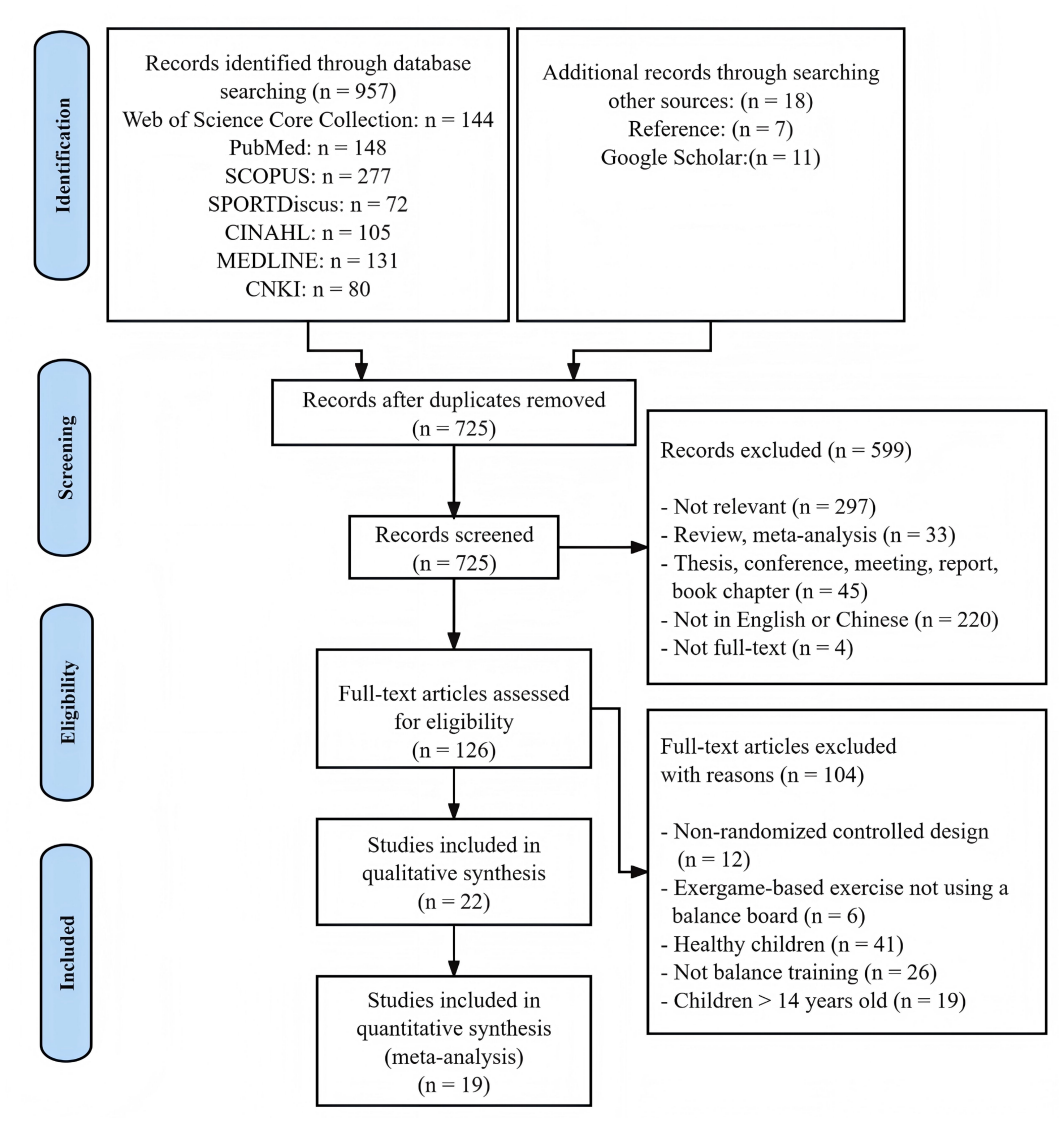
PRISMA flow diagram.

Figure 1 illustrates the systematic search process, which initially yielded 957 records from electronic databases, including PubMed, Web of Science Core Collection, CINAHL, MEDLINE, SPORTDiscus, Scopus, and CNKI. An additional 18 studies were identified through sources, such as Google Scholar and reference lists. Following duplicate removal, title and abstract screening, and full-text assessment, 22 studies were included in the review, of which 19 were included in the final meta-analysis.

### Methodological quality and risk of bias of the included trials

The PEDro scores of the included studies ranged from five to eight, indicating moderate to high methodological quality. Five studies were rated as moderate quality (score = 5), whereas 17 were classified as high quality (scores ranging from six to eight). Detailed methodological quality scores are presented in Table 1.

**Figure 2:**
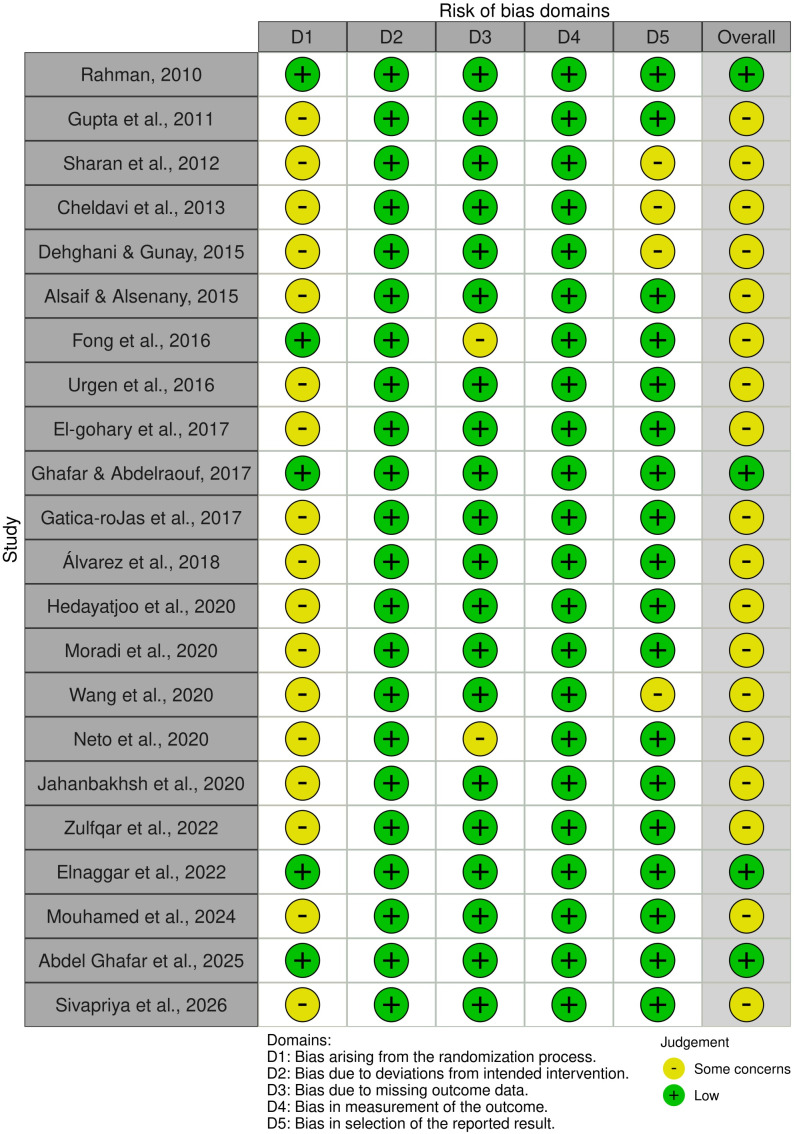
ROB-2 assessment traffic light plots.

**Figure 3:**
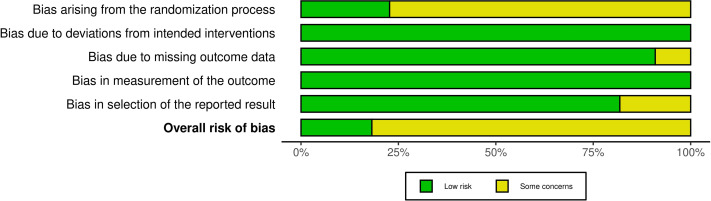
ROB-2 assessment weighted bar plots.

RoB 2 assessments were conducted for the included RCTs. Of these trials, four were judged to have a low risk of bias, while 18 showed some concerns, as illustrated in Figs. 2 and 3. Only five RCTs reported adequate methods for random sequence generation, whereas the randomization procedures in the remaining studies were insufficiently described. In addition, four trials presented some concerns related to participant selection, and two studies showed some concerns due to dropout rates exceeding 15%.

### Certainty of evidence

The GRADE assessment indicated that four outcome measures were of low certainty, with none rated as high certainty. Imprecision due to small sample sizes (90% of outcomes) was the primary reason for downgrading the quality of evidence. Two outcomes were automatically classified as having very low certainty (Table S1).

### Study characteristics

Table 2 outlines the characteristics of the RCTs included in the analysis. A total of 857 participants were involved across the 22 studies, consisting of 450 males, 243 females, and 155 participants with unspecified gender (Sharan et al., 2012; Dehghani & Gunay, 2015; AlSaif & Alsenany, 2015; Mouhamed et al., 2024). The reported age range of participants was 4 to 14 years. The participants had various conditions, including DS (Rahman & Rahman, 2010; Gupta, Rao & Kumaran, 2011; Wang et al., 2020; Ghafar & Abdelraouf, 2017; Álvareza et al., 2018), developmental coordination disorder (DCD) (Fong et al., 2016; Jahanbakhsh et al., 2020; Neto, Steenbergen & Wilson, 2025), CP (Sharan et al., 2012; AlSaif & Alsenany, 2015; Urgen et al., 2016; El-gohary et al., 2017; Gatica-rojas et al., 2017; Wang et al., 2020; Elnaggar et al., 2022; Mouhamed et al., 2024), ASD (Cheldavi et al., 2013; Abdel Ghafar et al., 2025), attention–deficit/hyperactivity disorder (ADHD) (Moradi, Jalali & Bucci, 2020), hearing impairment (HI) (Hedayatjoo et al., 2020), and intellectual disability (ID) (Dehghani & Gunay, 2015; Sivapriya, Punitha & Ahamed Ashwaq, 2026). In all studies, BT was either the sole intervention or a major component of the experimental protocol, while control groups participated in non-balance exercises, regular physical education classes, or received no intervention. Twelve studies employed exergame–based BT using the Wii Fit Balance Board, whereas ten RCTs implemented regular BT, such as static exercises (*e.g.*, single-leg standing), dynamic tasks (*e.g.*, weight shifting and walking-based balance activities), and training on unstable surfaces. Across the 22 included studies, participants completed one to three sessions per week, totaling 9–48 training sessions over intervention periods ranging from 3 to 16 weeks. A total of 19 studies specified the duration of each BT session (15–60 min), while three studies did not provide details on session length (Gupta, Rao & Kumaran, 2011; Sharan et al., 2012; Zulfiqar et al., 2022).

**Table 1:** Physiotherapy Evidence Database (PEDro) scale ratings.

**Study name**	**Eligibility criteria**	**Random allocation**	**Concealed allocation**	**Group similar at baseline**	**Blind subject**	**Blind therapist**	**Blind assessor**	**Follow -up**	**Intention- to-treat analysis**	**Between -group comparisons**	**Point measure and variability**	Total^*^	**Study quality**
Rahman & Rahman (2010)	1	1	0	1	0	0	0	1	1	1	1	6	High
Gupta, Rao & Kumaran (2011)	1	1	0	1	0	0	0	1	1	1	1	6	High
Sharan et al. (2012)	0	1	0	1	0	0	0	1	1	0	1	5	Moderate
Cheldavi et al. (2013)	1	1	0	1	0	0	0	1	1	1	1	6	High
Dehghani & Gunay (2015)	1	1	0	1	0	0	0	1	1	0	1	5	Moderate
AlSaif & Alsenany (2015)	1	1	0	1	0	0	0	1	1	0	1	5	Moderate
Fong et al. (2016)	1	1	1	1	0	1	0	0	1	1	1	7	High
Urgen et al. (2016)	1	1	0	1	0	0	0	1	1	1	1	6	High
Gatica-rojas et al. (2017)	1	1	0	1	0	0	0	1	1	1	1	6	High
El-gohary et al. (2017)	1	1	0	1	0	0	0	1	1	1	1	6	High
Ghafar & Abdelraouf (2017)	1	1	1	1	0	0	0	1	1	1	1	7	High
Álvareza et al. (2018)	1	1	0	1	0	0	0	1	1	1	1	6	High
Hedayatjoo et al. (2020)	1	1	0	1	0	0	0	1	1	1	1	6	High
Moradi, Jalali & Bucci (2020)	1	1	0	1	1	0	0	1	1	0	1	6	High
Wang et al. (2020)	1	1	0	1	0	0	0	1	1	0	1	5	Moderate
Jahanbakhsh et al. (2020)	1	1	0	1	0	0	0	1	1	1	1	6	High
Neto, Steenbergen & Wilson (2025)	1	1	0	1	0	1	1	0	1	1	1	7	High
Zulfiqar et al. (2022)	1	1	0	1	0	0	0	1	1	0	1	5	Moderate
Elnaggar et al. (2022)	1	1	1	1	0	0	0	1	1	1	1	7	High
Mouhamed et al. (2024)	1	1	1	1	1	0	0	1	1	1	1	8	High
Abdel Ghafar et al. (2025)	1	1	1	1	0	1	0	1	1	1	1	8	High
Sivapriya, Punitha & Ahamed Ashwaq (2026)	1	1	0	1	0	0	0	1	1	1	1	6	High

**Notes.**

A detailed explanation for each PEDro scale item can be accessed at https://www.pedro.org.au/english/downloads/pedro-scale.

* Item 1 is not included in the total score, which has a maximum possible value of 10.

### Results of synthesis

Given the nature of the intervention, all included studies (*n* = 22) identified balance as the primary outcome and assessed it using various measurement tools. Accordingly, separate meta-analyses were conducted for different balance domains across the included studies. Detailed information is presented in Table 3.

**Table 2:** Characteristics of included studies.

**Study**	**Participants**				**Training program**				**Outcomes**
	***n***	**Age (year)**	**Sex**	**Disorder**	**Intervention**	**Weeks**	**Freq**	**Time**	
Rahman & Rahman (2010)	30	10–13	M = 13 *F* = 17	DS	EG: Nintendo Wii Fit, CG: traditional physical therapy	6	2	15	Static and dynamic Balance (BOTMP)
Gupta, Rao & Kumaran (2011)	23	10–14	M = 14 *F* = 9	DS	EG: progressive resistive exercises and balance training; CG: regular activities	6	3	NR	Static and dynamic balance (BOTMP), Strength (hip flexors, hip abductors, hip extensors, knee flexors, knee extensors and ankle plantarflexors)
Sharan et al. (2012)	29	EG: 8.88 ± 3.23, CG: 10.38 ± 4.41	NR	CP	EG: VR training on a Wii balance board, CG: conventional training	3	3	NR	Functional balance (PBS)
Cheldavi et al. (2013)	20	7.90 ± 1.10	M	ASD	EG: balance training, CG: daily activities	6	3	45	Static balance (COP)
Dehghani & Gunay (2015)	22	10.23 ± 1.43	NR	ID	EG: balance training; CG: regular school schedule	10	2	40	Static balance (BOTMP), dynamic balance (BOTMP)
AlSaif & Alsenany (2015)	40	6–10	NR	CP	EG: Nintendo Wii Fit, CG: no training	12	1	20	Balance (BOTMP)
Fong et al. (2016)	130	6–10	M = 89 *F* = 41	DCD	EG1: functional movement (balance)–power training, EG2: functional movement (balance) training, CG: no physical training	12	2	30	Static balance (SOT), muscle strength (knee flexor, knee extensors)
Urgen et al. (2016)	30	7–14	M = 14 *F* = 16	CP	EG: Nintendo Wii balance board, CG: standard physiotherapy	9	2	45	Gross Motor Function Measure (GMFM), static balance (single leg standing), dynamic balance (TUG), functional balance (PBS)
El-gohary et al. (2017)	48	5–8	M = 26 *F* = 22	CP	EG: balance training, CG: traditional physical therapy	12	3	60	Functional balance (BBS) Gross motor skills (GMFM-88)
Ghafar & Abdelraouf (2017)	26	6–9	M = 18 *F* = 8	DS	EG: Wii balance board, CG: traditional physical therapy	8	3	30	Static balance (Five-times-sit-to-stand test), dynamic balance (TUG), functional balance (PBS)
Gatica-rojas et al. (2017)	32	10.7 ± 3.2	M = 19 *F* = 13	CP	EG: Nintendo Wii balance board, CG: standard physiotherapy	6	3	30	Static balance (COP)
Álvareza et al. (2018)	16	6–12	M = 13 *F* = 3	DS	EG: Wii Balance Board, CG: normal daily activities	5	2	20	Static balance (COP), gross motor development (TGMD-2)
Hedayatjoo et al. (2020)	36	7–12	M = 16 *F* = 20	HD	EG: balance training, CG: regular school schedule	4	3	45	Static and dynamic balance (BOTMP), coordination (upper limb, bilateral, visual motor control)
Moradi, Jalali & Bucci (2020)	30	8.58 ± 0.93	M	ADHD	EG1: balance training, CG: regular school schedule	7	3	30	Static balance (Biodex balance system), dynamic balance (Biodex balance system)
Wang et al. (2020)	50	7–12	M = 24 *F* = 26	CP	EG: balance training, CG: regular activities	16	3	30	Functional balance (BBS), static (DLO, DLC), dynamic balance (balance check system)
Neto, Steenbergen & Wilson (2025)	32	7–10	M = 24 *F* = 8	DCD	EG: Nintendo Wii balance board, CG: No-Wii task-specific training	8	2	60	Static and dynamic Balance (MABC-2), manual dexterity, aiming and catching
Jahanbakhsh et al. (2020)	39	7–9	M	DCD	EG1: balance training (single-task), EG2: balance training (dual-task), CG: no intervention	8	3	45	dynamic balance (Y Balance Test), static balance (Stork balance stand test)
Zulfiqar et al. (2022)	20	8.70 ± 3.23 7.30 ± 2.49	M = 12 *F* = 8	DS	EG: trunk balance exercises, CG: core stability exercises	6	3	NR	Functional balance (BBS)
Elnaggar et al. (2022)	57	8–12	M = 36 *F* = 21	CP	EG1: balance training, EG2: plyometric + balance training, CG: plyometric training	8	2	45	Muscle strength (quadriceps, hamstrings, ankle dorsiflexors), static balance (Biodex Stability System)
Mouhamed et al. (2024)	64	9–14	NR	CP	EG: VR training on a Wii balance board, CG: physical therapy	12	3	30	Static balance (Biodex Balance System), functional balance (PBS)
Abdel Ghafar et al. (2025)	53	7–12	M = 32 *F* = 21	ASD	EG: Nintendo Wii Balance Board and Wii Fit Plus, CG: traditional physical therapy	12	3	30	Static balance (BSS), functional balance (PBS)
Sivapriya, Punitha & Ahamed Ashwaq (2026)	30	6–12	M = 20 *F* = 10	ID	EG: exergaming with balance board, CG: conventional occupational therapy	12	3	45	Functional balance (PBS)

**Notes.**

M male F female Freq frequency EG experimental group CG control group VR virtual reality TGMD-2 Test of Gross Development BOTMP Bruininks-Oseretsky Test of Motor Proficiency SOT sensory organization test UST unilateral Stance Test MABC Movement Assessment Battery BBS Berg balance scale PBS Pediatric balance scale TUG the timed up and go test NR not reported DS Down syndrome CP Cerebral palsy ASD autism spectrum disorder DCD developmental coordination disorder ADHD attention-deficit/hyperactivity disorder COP center of pressure HD hearing deficit ID Intellectual disability DLO double-leg stance with eyes open DLC double-leg stance with eyes closed

**Table 3:** Classification of balance domains.

**Domain**	**Measurements**
Static Balance (*i.e.,* maintaining a posture)	Biodex Balance System (Moradi, Jalali & Bucci, 2020; Abdel Ghafar et al., 2025; Mouhamed et al., 2024; Elnaggar et al., 2022) Five Times Sit to Stand Test (Abdel Ghafar & Abdelraouf, 2017) Center of Pressure (Álvareza et al., 2018; Gatica-rojas et al., 2017; Cheldavi et al., 2013; Wang et al., 2020) Single Leg Standing (Urgen et al., 2016) Bruininks-Oseretsky Test of Motor Proficiency (Dehghani & Gunay, 2015) Sensory Organization Test (Fong et al., 2016) Stork Stand Test (Jahanbakhsh et al., 2020)
Dynamic Balance (*i.e.,* changing positions and moving through the environment)	Timed Up and Go Test (Abdel Ghafar & Abdelraouf, 2017; Urgen et al., 2016), Y Balance Test (Jahanbakhsh et al., 2020) Bruininks-Oseretsky Test of Motor Proficiency (Dehghani & Gunay, 2015), Biodex Balance System (Moradi, Jalali & Bucci, 2020) Balance Check System (Wang et al., 2020)
Functional Balance (*i.e.,* maintaining postural stability while performing goal-directed movements and functional tasks)	Pediatric Balance Scale (Abdel Ghafar et al., 2025; Abdel Ghafar & Abdelraouf, 2017; Mouhamed et al., 2024; Sharan et al., 2012; Urgen et al., 2016; Sivapriya, Punitha & Ahamed Ashwaq, 2026) Berg balance scale (El-gohary et al., 2017; Wang et al., 2020; Zulfiqar et al., 2022)

**Figure 4:**
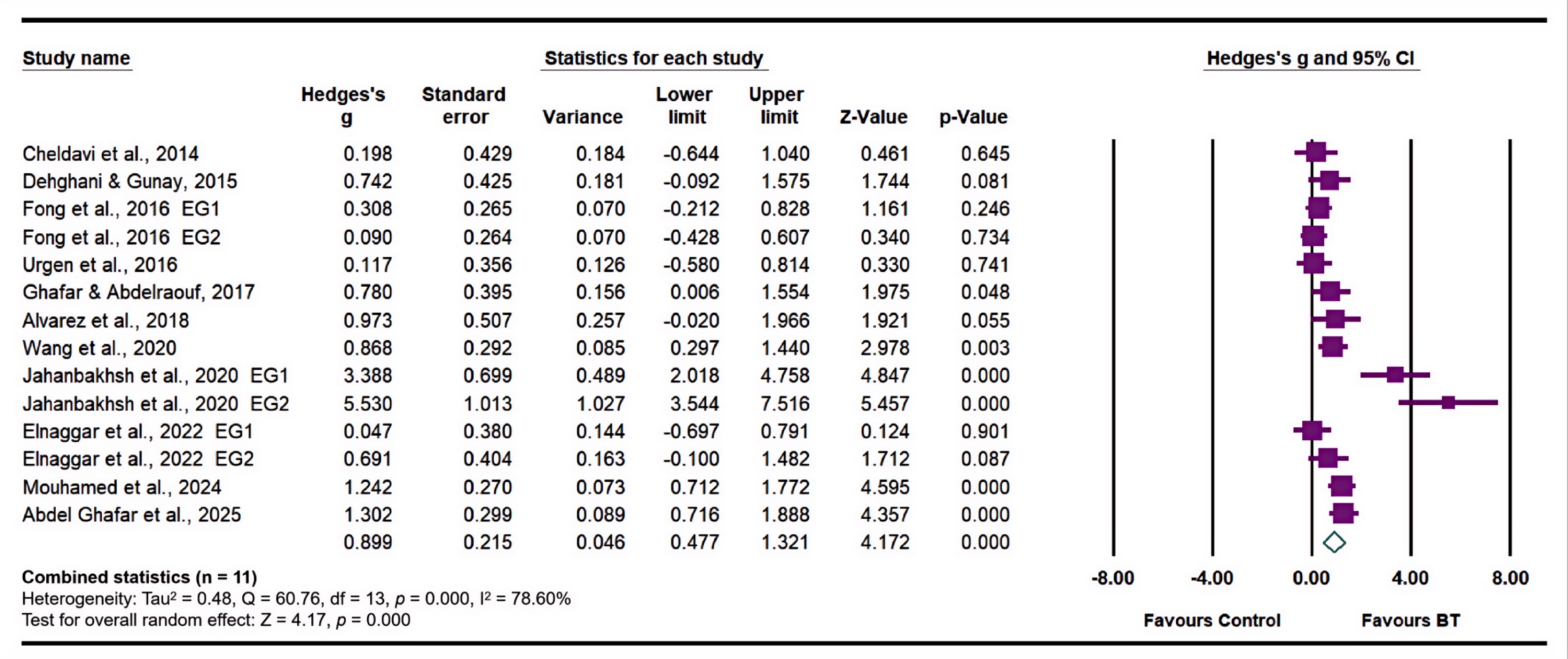
Static balance forest plot.

The balance outcomes across these studies provided sufficient data for meta-analysis. Eleven studies examining static balance showed moderate and significant improvements in the BT groups compared to controls (ES = 0.90; 95% CI [0.48–1.32]; *p* < 0.001; Fig. 4). A high heterogeneity (*I*^2^ = 78.60%) was observed. The funnel plot seemed asymmetrical (Fig. S1), and Egger’s test showed risk of publication bias (*p* = 0.028). After the trim and fill method, the adjusted values indicated a point estimate of ES = 0.76 (95% CI [0.28–1.24]) (Table S2). No significant sub-group difference (between-group *p* = 0.342) was found when BT interventions with ≤10 weeks (nine study groups; ES = 1.09; 95% CI [0.52–1.66]; within-group *I*^2^ = 83.36%) were compared to BT interventions with >10 weeks (five study groups; ES = 0.65; 95% CI [−0.04–1.34]; within-group *I*^2^ = 65.51%). No significant subgroup difference was found when exergame-based BT (five study groups; ES = 0.89; 95% CI = 0.17 to 1.61; within-group *I*^2^ = 51.34%) was compared with regular BT (nine study groups; ES = 0.91; 95% CI [0.37–1.59]; within-group *I*^2^ = 83.48%).

**Figure 5:**
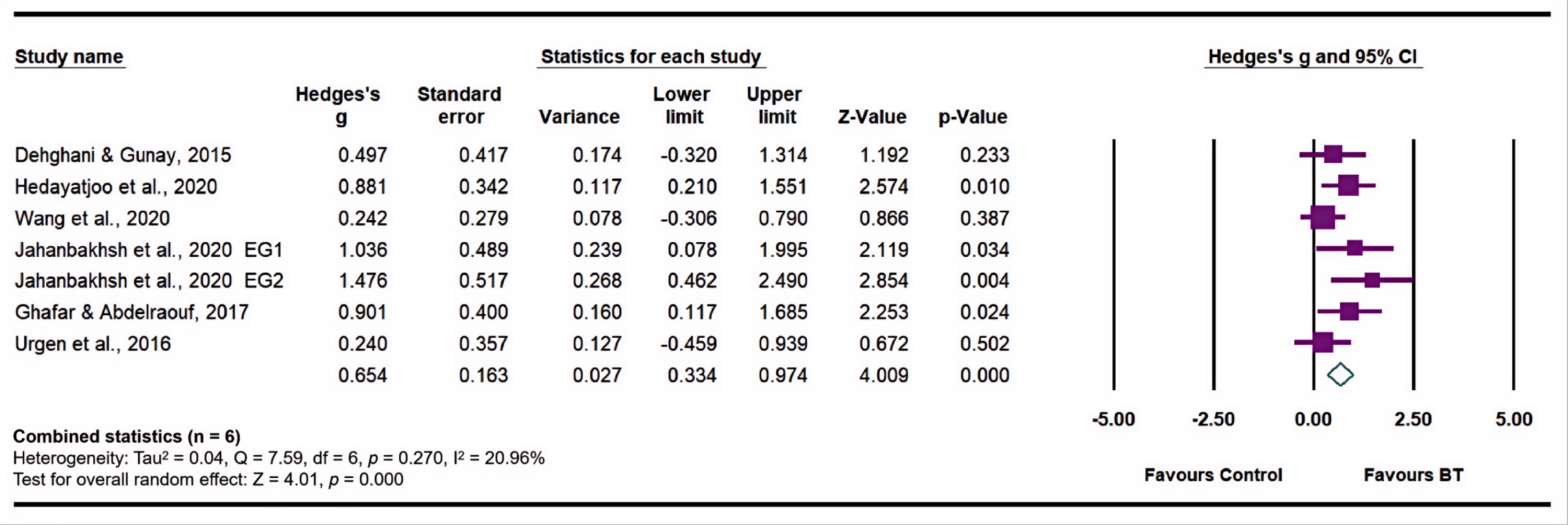
Dynamic balance forest plot.

Six studies examining dynamic balance show moderate and significant improvements in the BT groups compared to controls (ES = 0.65; 95% CI [0.33–0.97]; *p* < 0.001; Fig. 5). A low heterogeneity (*I*^2^ = 20.96%) was observed, and the Egger’s test indicated *p* = 0.038. After the trim and fill method, the adjusted values indicated a point estimate of ES = 0.52 (95% CI = 0.19–0.86) (Table S2).

**Figure 6:**
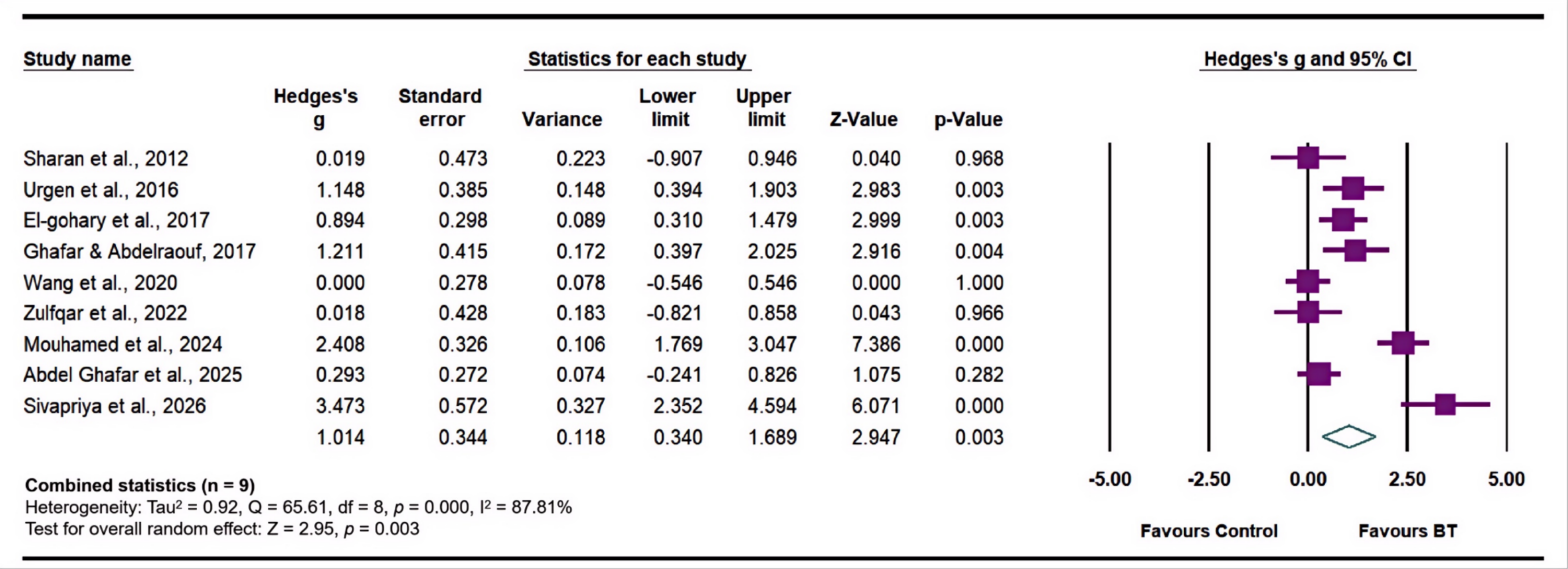
Functional balance forest plot.

Nine studies examining functional balance showed moderate and significant improvements in the BT groups compared to controls (ES = 1.01; 95% CI [0.34–1.69]; *p* = 0.003; Fig. 6). High statistical heterogeneity was present in the comparison (*I*^2^ = 87.81%), and the Egger’s test indicated *p* = 0.295. No significant sub-group difference (between–group *p* = 0.678) was found when BT interventions with ≤10 weeks (four study groups; ES = 0.61; 95% CI [−0.32–1.55]; within-group *I*^2^ = 59.74%) were compared to BT interventions with >10 weeks (five study groups; ES = 1.33; 95% CI [0.38–2.28]; within-group *I*^2^ = 92.98%). No significant subgroup difference was observed when exergame-based BT (six study groups; ES = 1.38; 95% CI [0.57–2.19]; within-group *I*^2^ = 89.33%) was compared with regular BT (three study groups; ES = 0.31; 95% CI [−0.64–1.27]; within-group *I*^2^ = 63.71%) (between-group *p* = 0.247).

**Figure 7:**
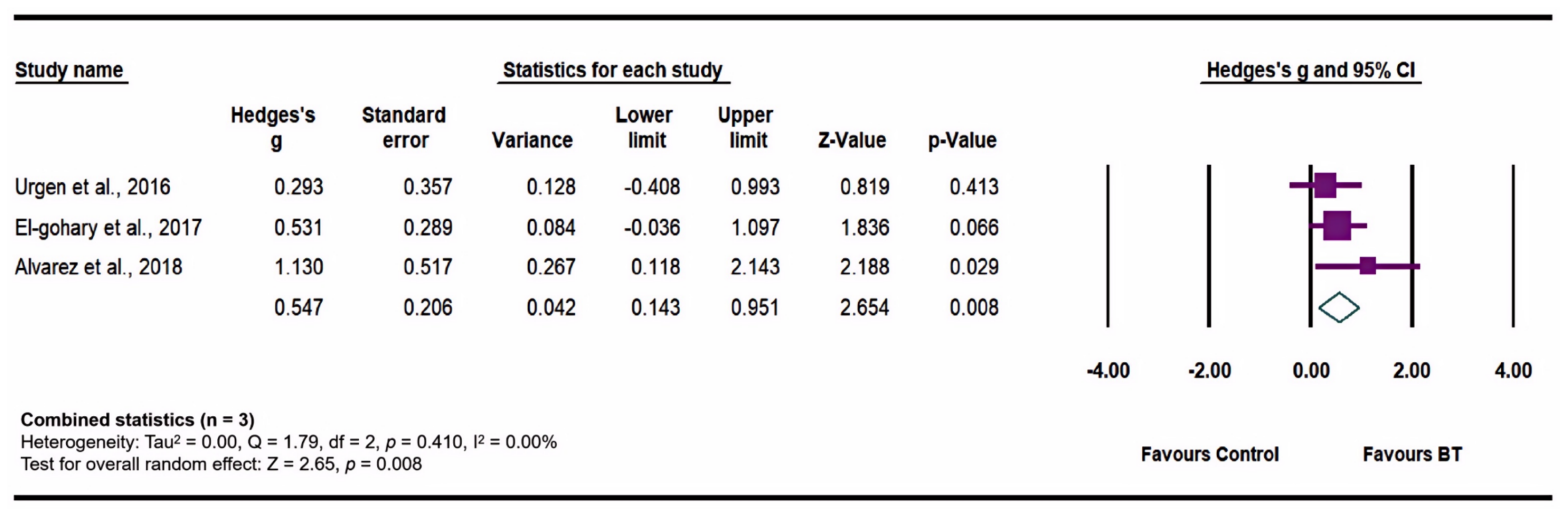
Gross motor function forest plot.

Three studies explored gross motor function (Urgen et al., 2016; El-gohary et al., 2017; Álvareza et al., 2018). Gross motor development was evaluated through the Test of Gross Development (TGMD) (Álvareza et al., 2018) and Gross Motor Function Measure (GMFM) (Urgen et al., 2016; El-gohary et al., 2017). The meta-analysis showed that moderate and significant improvements in the BT groups compared to controls (ES = 0.55; 95% CI [0.14–0.95]; *p* = 0.008; Fig. 7). A low heterogeneity (*I*^2^ = 0.00%) was observed, and the Egger’s test indicated *p* = 0.505.

Three studies (Gupta, Rao & Kumaran, 2011; Fong et al., 2016; Elnaggar et al., 2022) assessed strength outcomes and found significant improvements. Fong et al. (2016) examined children (6–10 years) with DCD, reporting that knee flexor peak force time improved at three months (−0.32 s) in the balance + power training group but declined by six months. In contrast, the balance–only group showed gains at six months (−0.15 s), though no differences emerged between groups. Knee extensor peak force increased at three months (0.63 kg) in the balance + power training group but was not sustained, with no notable changes in other measures (peak force of knee flexors and time to peak force of knee extensors). Gupta, Rao & Kumaran (2011) studied children with DS, assigning them to either an experimental group, which completed six weeks of progressive resistive lower-limb exercises and BT, or a control group following regular school routines. Compared with the control group, the experimental group demonstrated significantly greater post-training improvements in knee strength (1.39–2.54 lbs), hip strength (0.95–1.74 lbs), and ankle plantarflexor strength (0.38 lbs). In a study by Elnaggar et al. (2022), 57 children with spastic hemiplegic CP were assigned to one of three intervention groups: plyometric training, BT, or a combined program. Results indicated that the combined approach produced significantly greater gains in muscle strength than BT intervention alone (70.64 ± 4.93 N *vs.* 64.24 ± 7.31N).

In another study, a BT program consisting of three 45-minute sessions per week over four weeks was administered to children diagnosed with hearing deficits aged 7–12 years. This study demonstrated that BT resulted in significant improvements in upper-limb coordination (post-pre difference: 3.50 ± 1.25) and bilateral coordination (post-pre difference: 2.39 ± 1.38) in these children (Hedayatjoo et al., 2020).

## Discussion

### Main findings

The evidence suggests that BT may produce moderate improvements in balance (ES = 0.67–0.90), which is expected given that all included studies incorporated balance-specific exercises. Such balance improvements reflect enhanced neuromuscular coordination and a greater ability to maintain postural stability during both static postures and transitional movements, which are fundamental to motor development (Fong et al., 2018). Moreover, improved balance capacity may facilitate daily motor tasks such as climbing stairs, running, and changing body positions (Yiou, Hamaoui & Allali, 2018), which are often impaired in children with developmental disorders (MacDonald et al., 2017). Lesinski et al. (2015) reported the effectiveness of BT in improving static balance (moderate effect, standardized mean difference (SMD) = 0.44) and dynamic balance (small effect, SMD = 0.44) among individuals with moderately severe stroke. Similarly, Gebel et al. (2018) reported that BT produced medium to large effects on static (SMD = 0.71) and dynamic balance (SMD = 1.03) in healthy youth, independent of training setting, training status, assessment method, age, or sex.

Several individual studies also support the positive effects of physical exercise on balance in individuals with disorders. For instance, Jankowicz-Szymanska, Mikolajczyk & Wojtanowski (2012) demonstrated that a three-month sensorimotor training program improved static balance in young individuals with mild DS. Likewise, Alsakhawi & Elshafey (2019) demonstrated that core stability exercises combined with treadmill training enhanced balance in children with DS. Gupta, Rao & Kumaran (2011) reported similar improvements following six weeks of BT. However, Zulfiqar et al. (2022) found that core stability exercises were more effective than BT in improving postural control in children with DS over a six-week period. This difference may be attributed to the role of trunk muscle activation preceding lower-extremity movements, which stabilizes the spine and supports purposeful movement. Giagazoglou et al. (2013) found that the transverse abdominis is activated prior to limb movement, reflecting a preprogrammed action controlled by the central nervous system to maintain spinal stability.

Existing literature indicates that children with ASD commonly exhibit deficits in postural control (Lim et al., 2017; Hariri et al., 2022). Two studies of the present review suggested that BT resulted in improvements of postural control in children with ASD (Cheldavi et al., 2013; Abdel Ghafar et al., 2025). Evidence from empirical studies suggests that high rates of postural sway in children with ASD were partially due to poor integration of vestibular, somatosensory, and visual inputs (Memari et al., 2013; Doumas, McKenna & Murphy, 2016). This deficit may lead to difficulties in maintaining an upright posture. In addition, neurobehavioural studies have demonstrated that the cerebellum and basal ganglia, which play established roles in postural control, show structural or functional impairments in these children (Date, Munn & Frey, 2024). After BT, children were able to perform the stance blindfolded or on the surface of a foam pad, suggesting improved balance and potentially enhanced function of the cerebellum and basal ganglia.

Interestingly, evidence from ten trials suggests that interventions employing the Nintendo Wii Balance Board may offer potential benefits for improving balance in children with developmental disorders (*e.g.*, DCD, DS), and subgroup analyses indicated no significant difference between exergame-based BT and conventional BT. This finding may be partly explained by the role of task repetition in balance exercises, which is central to neural reorganization and brain plasticity (Eggenberger et al., 2016). Exergames are inherently engaging and may enhance motivation, thereby promoting greater exercise repetition and potentially increasing training intensity (Davis et al., 2024). In contrast, Ramstrand & Lygnegård (2012) reported that Nintendo Wii Fit Balance Board training, performed for at least 30 min per day at home over a five-week period, was not effective in improving balance in children with hemiplegic or diplegic CP. In addition, eight of the included trials focused on children with CP and reported positive effects of BT in this review. Children with CP often present with impaired postural control that contributes to gait abnormalities (Dewar, Love & Johnston, 2015; Zhou, Butler & Rose, 2017). BT may enhance postural stability by promoting multisensory integration and improving neuromuscular coordination, thereby facilitating more efficient anticipatory and reactive postural adjustments (Surgent et al., 2019). Overall, childhood is characterized by high central nervous system plasticity, during which sensory systems underlying postural control mature sequentially and reach full development by approximately 15–16 years of age (Steindl et al., 2006). Therefore, implementing postural balance rehabilitation during this period is particularly effective, as the developing nervous system is highly responsive to multisensory stimulation.

However, high heterogeneity (I^2^ > 75%) was observed for both static and functional balance outcomes, which may substantially affect the interpretation of the pooled effect estimates. Several plausible sources may explain this variability. First, the included studies targeted heterogeneous populations with distinct neurodevelopmental disorders (*e.g.*, ID, DCD, ADHD, CP, DS, ASD, HI), each characterized by different balance deficits and neuromotor constraints, which may lead to differential responsiveness to BT. Second, substantial variation existed in balance assessment tools (*e.g.*, scales *versus* field–based functional tests), potentially capturing different constructs of balance control. Third, training protocols varied markedly in duration (3–16 weeks), frequency (2–3 sessions/week), and exercise content, all of which are known to influence training adaptations. Collectively, these methodological and clinical differences likely contributed to the observed heterogeneity; therefore, the findings should be interpreted with caution.

The meta-analysis pointed out that BT may significantly improve gross motor function in children with developmental disorders. Improvements in gross motor function may increase children’s participation in physical activity, play, sports, and school physical education (Lucas et al., 2016). Existing studies consistently suggest that BT improves postural alignment, enhances spinal control, and provides better proprioceptive feedback, which significantly enhances postural stability (Taube, Gruber & Gollhofer, 2008; Wiesmeier et al., 2017; Surgent et al., 2019), and postural stability serves as a foundation for gross motor skills (Mache & Todd, 2016). BT also promotes neuroplasticity, optimizing the central nervous system’s integration and regulation of posture and movement (Surgent et al., 2019; Rogge et al., 2018), thus providing more effective motor control strategies for dynamic tasks like standing and walking. Moreover, functional muscle strength is closely related to gross motor abilities (Verschuren et al., 2009), and the challenge of core and lower-limb stability in BT serves as a low-dose, repetitive, and function-oriented form of strength training (Hedayatjoo et al., 2020). However, due to the limited number of relevant studies (*n* = 3), no definitive conclusions can be drawn, and further high-quality RCTs are needed for verification.

### Additional evidence

Balance exercise has preliminary empirical support for improving muscle strength in children with DCD and DS (Gupta, Rao & Kumaran, 2011; Fong et al., 2016). Children with DCD often exhibit deficits in neuromuscular coordination and proprioceptive input, while those with DS experience limited muscle strength generation efficiency due to hypotonia and ligamentous laxity (Fong et al., 2018; Koo et al., 2022). Gupta, Rao & Kumaran (2011) found that after six weeks of exercise training, children with DS showed significant improvements in lower limb strength compared with a control group, suggesting that short-term interventions can elicit positive strength adaptations. The authors noted that due to the short duration, changes in muscle strength were likely due to enhanced neural recruitment rather than muscle fiber hypertrophy, which typically requires at least 12 weeks of training. Fong et al. (2016) demonstrated that children with DCD showed significant improvements in knee muscle strength after 12 weeks of BT or a combined balance and power training program. The balance + explosive training group exhibited shorter peak knee flexor force generation time and greater extensor peak force, with the effects of the balance group maintained after six months. Similar to other forms of strength training, BT enhances neural drive by improving motor unit recruitment, firing frequency, and synchronization (Morrison & Newell, 2023), particularly in stabilizing muscles. In addition, training under unstable or perturbed conditions increases joint co-contraction and muscle activation, while challenging proprioceptive and reflex pathways (Bao, Wang & Li, 2025), thereby promoting neural adaptations that improve force transmission and intramuscular coordination (Taube, 2012).

Of note, one study in the review compared three eight-week interventions–BT, plyometric training, and a combined approach, and found that the combined method more effectively improved muscle strength in children with spastic hemiplegic CP (Elnaggar et al., 2022). This outcome may be explained by the preconditioning effect, whereby neuromuscular adaptations resulting from BT enhance force production and postural stability, thereby improving the efficiency of plyometric movements (Bruhn, Kullmann & Gollhofer, 2006; Hammami et al., 2016). At the neuromuscular level, this may involve greater activation of knee joint and ankle muscles, further enhancing the effects of training (Ramezani, Saki & Tahayori, 2024). In line with this, several studies have indicated that combining strength training with BT positively impacts muscle strength (Zouita et al., 2020; Aartolahti et al., 2020). Additionally, a scoping review by Granacher & Behm (2023) concluded that six to ten weeks of combined resistance exercise and BT significantly improve balance and muscular fitness in healthy youth and athletes. However, the findings are primarily based on a small number of experimental studies, and their broad applicability and training effectiveness require further validation in future research.

Currently, only one study has examined the effects of a four-week BT program on coordination in children with HI, focusing on visual-motor control, bilateral coordination, and upper-limb coordination (Hedayatjoo et al., 2020). From a neurophysiological perspective, BT enhances the central nervous system’s integration and regulation of different body parts, particularly by improving proprioception and spatial awareness (Rogge et al., 2017). Children with HI often experience sensory integration deficits and balance dysfunction, which may indirectly affect upper-limb coordination and visual-motor integration (Gheysen, Loots & Van Waelvelde, 2008).

## Limitations and Future Directions

Several limitations of this review should be acknowledged, which also inform directions for future research and practice. First, due to the limited number of trials, subgroup analyses for several potential moderators, such as disorder type (*e.g.*, CP, DS, DCD, ASD), measurement instruments, and session duration, could not be conducted. This constraint reduces statistical power and underscores the need for further well-designed trials with larger sample sizes. Second, high heterogeneity (*I*^2^ = 78.60–87.81%) was observed across outcomes (*i.e.,* static and functional balance), likely due to variations in participant characteristics, intervention protocols, and assessment methods, which may reduce the robustness of the pooled estimates. Third, relatively few studies assessed broader motor performance domains beyond balance, such as muscle strength, coordination, and gross motor function, resulting in a narrow evaluative scope. Future trials should therefore adopt multidimensional assessment frameworks to capture the transfer effects of BT on overall motor competence and functional performance. Fourth, the evidence base was weighted toward children with CP (*n* = 8) and DS (*n* = 5), with limited representation of other neurodevelopmental conditions (*e.g.*, ASD, DCD, ADHD) and an absence of studies involving other types of developmental impairments. Future research should extend BT interventions to a wider range of developmental populations to improve external validity and to determine whether intervention effects differ across diagnostic groups. In addition, future research should prioritize the feasibility and scalability of BT in real-world clinical and educational settings by systematically examining whether key intervention characteristics (*e.g.*, session intensity, duration, and level of therapist involvement) are transferable from controlled research protocols to routine practice.

## Conclusions

The available evidence suggests that BT programs lasting 3–16 weeks, delivered 2–3 sessions per week with session durations of 15–60 min, may be associated with improvements in balance in children with developmental disorders. Evidence for improvements in gross motor function is based on a small number of trials and should therefore be interpreted cautiously. Although only a limited number of studies have examined outcomes such as muscle strength and coordination, preliminary findings indicate potential positive trends in these domains. Nevertheless, the current evidence base is constrained by methodological heterogeneity, and evidence-based guidance on optimal BT protocols (*e.g.*, training modalities, intensity, and progression) remains lacking. Future research should prioritize high-quality, standardized trials that investigate task-specific BT interventions (*e.g.*, cognitive-motor dual-task designs) and systematically examine dose–response relationships to better clarify effectiveness and support translation to real-world clinical and educational settings.

##  Supplemental Information

10.7717/peerj.21272/supp-1 Supplemental Information 1 Search strategy

10.7717/peerj.21272/supp-2 Supplemental Information 2 GRAND table and funnel plot

10.7717/peerj.21272/supp-3 Supplemental Information 3 The dataset used for meta-analysis

10.7717/peerj.21272/supp-4 Supplemental Information 4 PRISMA checklist

## Additional Information and Declarations

### Data Availability

The following information was supplied regarding data availability: This is a systematic review/meta-analysis. The raw data is available in the Supplemental File.
