# Supplementary material for: Systematic review and meta-analysis of balance training in children with developmental disorders
Source: PeerJ. 2026 Jun 10;14:e21272. doi: 10.7717/peerj.21272 (PMC13264278; doi:10.7717/peerj.21272)
Supplement: Supplemental Information 1 [file peerj-14-21272-s001.docx]

**Detailed search strategy**

**Search on 11 January 2026**

| **Databases** | **search strategy** | **Results** |
| --- | --- | --- |
| PubMed  (https://pubmed.ncbi.nlm.nih.gov/) | (("balance training"[Title/Abstract] OR "balance exercise*"[Title/Abstract] OR "instability training"[Title/Abstract] OR "perturbation training"[Title/Abstract] OR "proprioceptive training"[Title/Abstract] OR "sensorimotor training"[Title/Abstract] OR "Wii Fit"[Title/Abstract] OR "balance board"[Title/Abstract]) AND (disabilit*[Title/Abstract] OR disorder*[Title/Abstract] OR deficit[Title/Abstract] OR "cerebral palsy"[Title/Abstract] OR "Down Syndrome"[Title/Abstract] OR "autism spectrum[Title/Abstract])) AND (children[Title/Abstract] OR puberty[Title/Abstract] OR kids[Title/Abstract] OR teen*[Title/Abstract] OR boy*[Title/Abstract] OR girl*[Title/Abstract]) | 148 |
| Web of Science Core Collection (https://www.webofscience.com/wos/alldb/basic-search) | (AB=(“balance training” OR “balance exercise*” OR “instability training” OR “perturbation training” OR “proprioceptive training” OR “sensorimotor training” OR “Wii Fit” OR “balance board”) AND AB=(disabilit* OR disorder* OR deficit OR “cerebral palsy” OR “Down Syndrome” OR “autism spectrum”) AND (children OR puberty OR kids OR teen* OR boy* OR girl*) | 144 |
| SPORTDicus (via EBSCOhost) (https://about.ebsco.com/products/research-databases/sportdiscus-full-text) | XB (“balance training” OR “balance exercise*” OR “instability training” OR “perturbation training” OR “proprioceptive training” OR “sensorimotor training” OR “Wii Fit” OR “balance board”) AND XB (disabilit* OR disorder* OR deficit OR “cerebral palsy” OR “Down Syndrome” OR “autism spectrum”) AND XB (children OR puberty OR kids OR teen* OR boy* OR girl*) | 72 |
| Scopus  (https://www.elsevier.com/products/scopus) | ( TITLE-ABS-KEY ( balance training” OR “balance exercise*” OR “instability training” OR “perturbation training” OR “proprioceptive training” OR “sensorimotor training” OR “Wii Fit” OR “balance board”) AND TITLE-ABS-KEY ( disabilit* OR disorder* OR deficit OR “cerebral palsy” OR “Down Syndrome” OR “autism spectrum”) AND TITLE-ABS-KEY (children OR puberty OR kids OR teen* OR boy* OR girl*) | 277 |
| CINAHL (via EBSCOhost) (https://about.ebsco.com/products/research-databases/cinahl-database) | XB (“balance training” OR “balance exercise*” OR “instability training” OR “perturbation training” OR “proprioceptive training” OR “sensorimotor training” OR “Wii Fit” OR “balance board”) AND XB (disabilit* OR disorder* OR deficit OR “cerebral palsy” OR “Down Syndrome” OR “autism spectrum”) AND XB (children OR puberty OR kids OR teen* OR boy* OR girl*) | 105 |
| MEDLINE (via EBSCOhost) (https://about.ebsco.com/products/research-databases/medline) | XB (“balance training” OR “balance exercise*” OR “instability training” OR “perturbation training” OR “proprioceptive training” OR “sensorimotor training” OR “Wii Fit” OR “balance board”) AND XB (disabilit* OR disorder* OR deficit OR “cerebral palsy” OR “Down Syndrome” OR “autism spectrum”) AND XB (children OR puberty OR kids OR teen* OR boy* OR girl*) | 131 |
| China National Knowledge Infrastructure (CNKI) (https://www.cnki.net/) | 平衡训练（balance training） AND 儿童（children） | 80 |
| Total |  | 957 |

Note: Because Chinese search terminology is relatively standardized and fixed in usage, we restricted the Chinese database （CNKI）search to two core terms only, namely “平衡训练”（balance training） AND “儿童”（children）, to ensure comprehensive yet consistent retrieval of relevant studies.
