# Supplementary material for: Systematic review and meta-analysis of balance training in children with developmental disorders
Source: PeerJ. 2026 Jun 10;14:e21272. doi: 10.7717/peerj.21272 (PMC13264278; doi:10.7717/peerj.21272)
Supplement: Supplemental Information 2 [file peerj-14-21272-s002.docx]

| **Table S1. GRADE analyses** | | | | | | | |
| --- | --- | --- | --- | --- | --- | --- | --- |
| **Outcomes** | **Certainty assessment** | | | | | **No of participants and studies** | **Certainty of evidence (GRADE)** |
|  | **Risk of bias** | **Inconsistency** | **Indirectness** | **Imprecision** | **Risk of publication bias** |  |  |
| Static balance  follow-up: range 5 to 12 weeks | Not Serious | Serious ^b^ | Not serious | Not serious | Serious ^c^ | 485 (11 RCTs) | ⨁⨁◯◯  LOW |
| Dynamic balance  follow-up: range 7 to 16 weeks | Not Serious | Not serious | Not serious | Serious ^c^ | Serious ^c^ | 203 (6 RCTs) | ⨁⨁◯◯  LOW |
| Functional balance  follow-up: range 3 to 12 weeks | Not Serious | Serious ^b^ | Not serious | Serious ^c^ | Not serious | 337 (9 RCTs) | ⨁⨁◯◯  LOW |
| Gross motor function  follow-up: range 5 to 12 weeks | Serious ^a^ | Not serious | Not serious | Serious ^c^ | Not serious | 94 (3 RCTs) | ⨁⨁◯◯  LOW |
| Strength  follow-up: range 6 to 12 weeks | - | - | - | - | - | 210 (3 RCTs) | ⨁◯◯◯  VERY LOW |
| Coordination  follow-up: 4 weeks | - | - | - | - | - | 36 (1 RCT) | ⨁◯◯◯  VERY LOW |

GRADE, Grading of Recommendations Assessment, Development and Evaluation; RCTs, Randomized controlled trials

a Downgraded by one level due to average PEDro score being moderate (< 6)

b Downgraded by one level due to the high impact of statistical heterogeneity (> 75%)

c Downgraded by one level, as < 400 participants were available for a comparison or a wide confidence interval (CI) around the effect estimate; we considered a CI to be

wide if it included both a small (0.2-0.6) and large effect size (> 1.2-2.0). Downgraded by two levels in case of imprecision based on both assessed points.

**Figure S1. Funnel Plot for Static Balance**


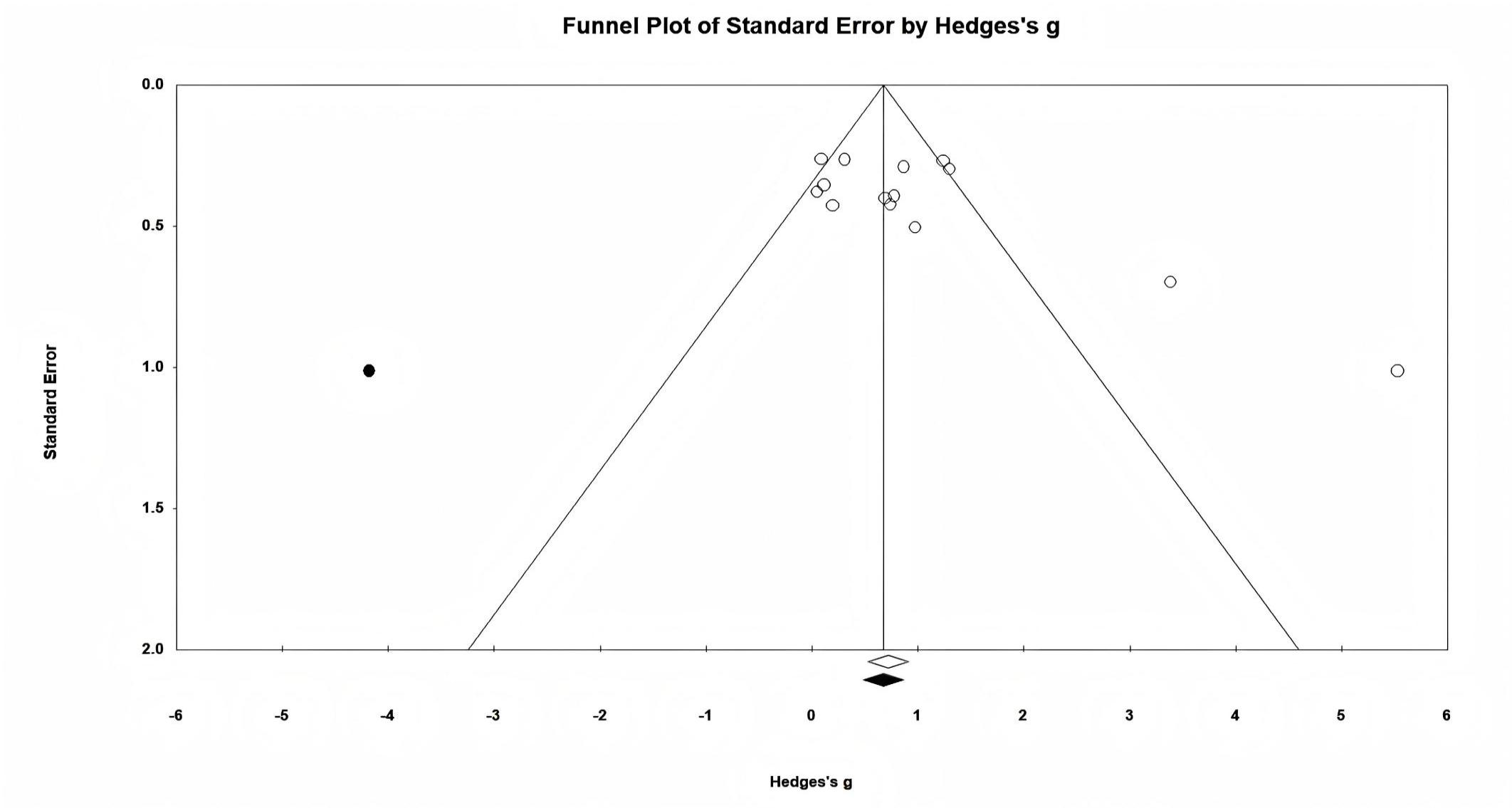


Note: the white dots represent the observed studies, while the black dots indicate the imputed studies that were added to correct for asymmetry.

**Table S2.** Values of Before and After Trim-and-Fill

| Outcomes | Before Trim-and-Fill | | | After Trim-and-Fill | | |
| --- | --- | --- | --- | --- | --- | --- |
|  | ES | 95%CI | Q value | ES | 95%CI | Q value |
| Static balance | 0.90 | 0.48–1.32 | 60.76 | 0.76 | 0.28–1.24 | 83.91 |
| Dynamic balance | 0.65 | 0.33–0.97 | 7.59 | 0.52 | 0.19–0.86 | 12.93 |

Note: ES, effect size; CI, Confidence interval.
